# Cutaneous Leukemic Infiltrates Successfully Treated With Biomodulatory Therapy in a Rare Case of Therapy-Related High Risk MDS/AML

**DOI:** 10.3389/fphar.2018.01279

**Published:** 2018-11-13

**Authors:** Daniel Heudobler, Sebastian Klobuch, Simone Thomas, Joachim Hahn, Wolfgang Herr, Albrecht Reichle

**Affiliations:** Department of Internal Medicine III, University Hospital Regensburg, Regensburg, Germany

**Keywords:** leukemic skin infiltration, myelodysplastic syndrome, acute myeloid leukemia, biomodulatory treatment, anakoinosis

## Abstract

Cutaneous manifestations in hematologic malignancies, especially in leukemia, are not common and may be very variable. Here we report a very unusual case of a patient (female, 70 years old) who was admitted to the hospital in 2016 because of skin lesions on the face, the trunk of the body and the extremities. She had a history of breast cancer in the year 2004 (pT1b, pN0, cM0, L0, V0, R0) which had been resected and treated with adjuvant radiation and chemotherapy (cyclophosphamide, methotrexate, 5-fluorouracile) as well as psoriasis treated with methotrexate and cyclosporine. Because of mild cytopenia a bone marrow aspirate/biopsy was performed showing myelodysplastic syndrome (MDS) with multilineage dysplasia. Cytogenetic review revealed a complex aberrant karyotype denoting adverse outcome. Simultaneously, a skin biopsy could confirm leukemic skin infiltration. Consequently, a therapy with azacitidine was started. After the first cycle the patient developed severe pancytopenia with a percentage of 13% peripheral blasts (previously 0–2%) as well as fever without evidence for infection which was interpreted as progressive disease. Therefore, the therapeutic regimen was changed to a biomodulatory therapy consisting of low-dose azacitidine 75 mg/day (given sc d1-7 of 28), pioglitazone 45 mg/day per os, and all-trans-retinoic acid (ATRA) 45 mg/m2/day per os. After cycle 1 of this combined biomodulatory therapy the patient showed hematologic recovery; besides a mild anemia (hemoglobin 11.1 g/dl) she developed a normal blood count. Moreover, the cutaneous leukemic infiltrates which had been unaffected by the azacitidine ameliorated tremendously after 2 cycles resulting in a complete remission of the skin lesions after cycle 6. In conclusion, we report a very unusual case with cutaneous infiltrates being the first clinical manifestation of hematologic disease, preceding the development of acute myeloid leukemia. While azacitidine alone was ineffective, a combined biomodulatory approach resulted in a complete remission of the cutaneous manifestation.

## Introduction

### Clinical presentation

In May 2016 a 70-year-old female patient was admitted to the hospital suffering from multiple skin lesions which primarily appeared on the chest, sacrum and the face 3 months ago. Topic as well systemic therapy with (high dose) corticosteroids had been ineffective in the treatment. The patient also complained about pain of the big joints (knees, hips). Fever, night sweats or weight loss were denied. A skin biopsy had already been performed showing no evidence for malignancy or autoimmune disease.

**Figure 1:**
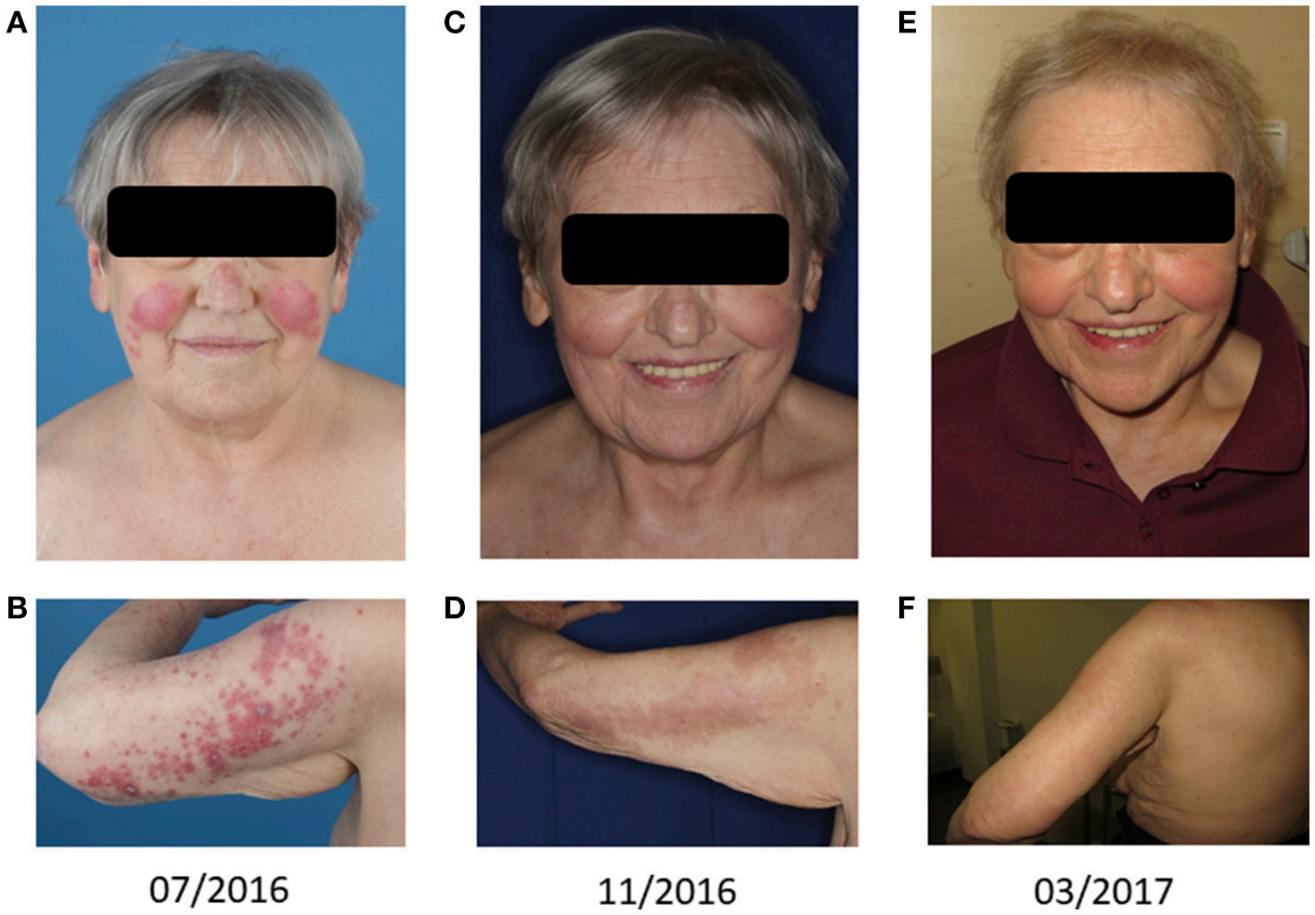
Cutaneous leukemic skin infiltrates: Skin lesions on the face **(A,C,E)** and the back of the left upper arm **(B,D,F)** are shown. Panels **(A,B)** represent the treatment-naive state. Panels **(C,D)** show the lesions after one cycle of azacitidine and one cycle of combined biomodulatory therapy. Panels **(E,F)** demonstrate complete remission of skin lesions after six cycles of biomodulatory treatment.

Physical examination revealed generalized erythematous infiltrated papules on the entire integument, multiple ulcers on both breasts and the back as well as papular erythema on both cheeks (Figures 1A,B). A broad laboratory work-up showed no pathologies besides mild leukopenia (leukocytes 3.8/nl), mild anemia (11 g/dl), and a positive ANA titer (1:160). Re-biopsy of the skin on the right upper arm demonstrated interface dermatitis consistent with the diagnosis of erythema exsudativum, drug rash or dermatomyositis. Chest x-ray and ultrasound of the abdomen showed no pathological findings. A therapy with topic agents as well as systemic corticosteroids (prednisolone 1 mg/kg) was (re-)started leading to minimal improvements.

## Background

### Medical history

In 2004, the patient had been diagnosed with papillary breast cancer of the left breast [pT1b, pN0 (0/8), sN0 (0/3), cM0, L0, V0, R0] which had been treated with breast conserving surgery, adjuvant radiation as well as adjuvant chemotherapy (CMF regimen; 6 cycles of cyclophosphamide, methotrexate, 5-fluorouracile). Periodic aftercare examinations showed no evidence for metastatic disease. Due to suspected dermatomyositis a CT-scan of the thorax and abdomen as well as a bone scintigraphy were performed in April 2016 without any pathological finding. Moreover, the patient has a history of psoriasis vulgaris since 1984, which had been treated with methotrexate and cyclosporine. Because of arthrosis, the patient received joint replacement surgery of both knees and the right hip joint.

## Discussion

### Diagnosis

Within the next 3 months, the skin lesions showed no improvement upon therapy. Since the laboratory work-up still revealed mild cytopenia (leukocytes 2.8/nl, hemoglobin 10.7 g/dl, MCV 81 fl, thrombocytes 153/nl) a bone marrow biopsy was performed in July 2016 confirming myelodysplastic syndrome with multilineage dysplasia and ringsideroblasts (MDS-RS-MLD): hypercellular marrow with dysplasia in all lineages and 36% ringsideroblasts. Bone marrow blasts (CD34+) were not increased. Because of the preceding chemo-/radiotherapy, the diagnosis therapy-related MDS (t-MDS) was stated. The genetic review revealed a complex aberrant karyotype involving chromosomes 5, 7, 8, 12, 13, 18, 21, and 22 (Karyotype formula: 45,XX,der(5;7)(5pter->5p11::7q22->7q11::5p11->5q11::7q11->7pter)[4]; 45,XX,der(5;7)(5pter->5p11::7q22->7q11::5p11->5q11::7q11->7pter),der(12;18)t(12;18)(p13;q23)del(12)(p12p13)[2]; 45,XX,der(5;7)(5pter->5p11::7q22->7q11::5p11->5q11::7q11->7pter),+8,der(12;18)t(12;18)(p13;q23)del(12)(p12p13),ider(13)(q10)del(13)(q13q22),+21,+22[4]; 46,XX[3]; ISCN: nuc ish 7cen(D7Z1x2),7q31(D7S486x1),8cen(D8Z2x3), 12p13(3′ETV6x1,5′ETV6x2)(3′ETV6 con 5′ETV6x1)[74/100]). Despite positive ringsideroblasts, no mutation in *SF3B1* could be found. IPSS-R (Revised International Prognostic Scoring System) (Greenberg et al., 2012) demonstrated high risk disease denoting adverse outcome.

Simultaneously, another skin biopsy was performed. Histological work-up in the reference center revealed mixed perivascular infiltrates consisting of lymphocytes and blasts with a high proliferation rate based on Ki67 staining; immuno-histochemically positivity of the blasts for myeloperoxidase (MPO) and KiM1P could be confirmed while being negative for CD34 (IHC panel: staining for CD3, CD20, CD34, MPO, KiM1P). In conclusion, leukemic skin infiltration could be confirmed.

Leukemic skin infiltrates, also termed leukemic skin (LC), are defined by infiltrates of the epidermis, dermis, or subcutis by neoplastic leukocytes (leukemia cells), resulting in clinically identifiable cutaneous lesions. LCs occur in ~3% of patients with acute myeloid leukemia (AML) (Agis et al., 2002), and less frequently in MDS (Patel et al., 2012); with most frequent association in AML with acute myelomonocytic and monocytic differentiation (involvement in up to 50% of patients) (Kaddu et al., 1999; Pont et al., 2001). Clinical manifestations vary from erythematous papules to plum-colored plaques and nodules that may become purpuric and ulcerate. Skin infiltrates can develop simultaneously, following, or rarely preceding the onset of systemic leukemia (Su, 1994). The latter, termed “aleukemic leukemia cutis,” occurs most often in patients who eventually develop AML. Patients with suspected LC should always undergo biopsy. But like in our case the diagnosis of LC can be challenging: Immuno-histochemistry can be very helpful in the process with MPO resembling a strong marker for myeloid cells while CD34 or CD117 remain very often negative (Traweek et al., 1993; Cronin et al., 2009; Li et al., 2018).

### Treatment

**Figure 2:**
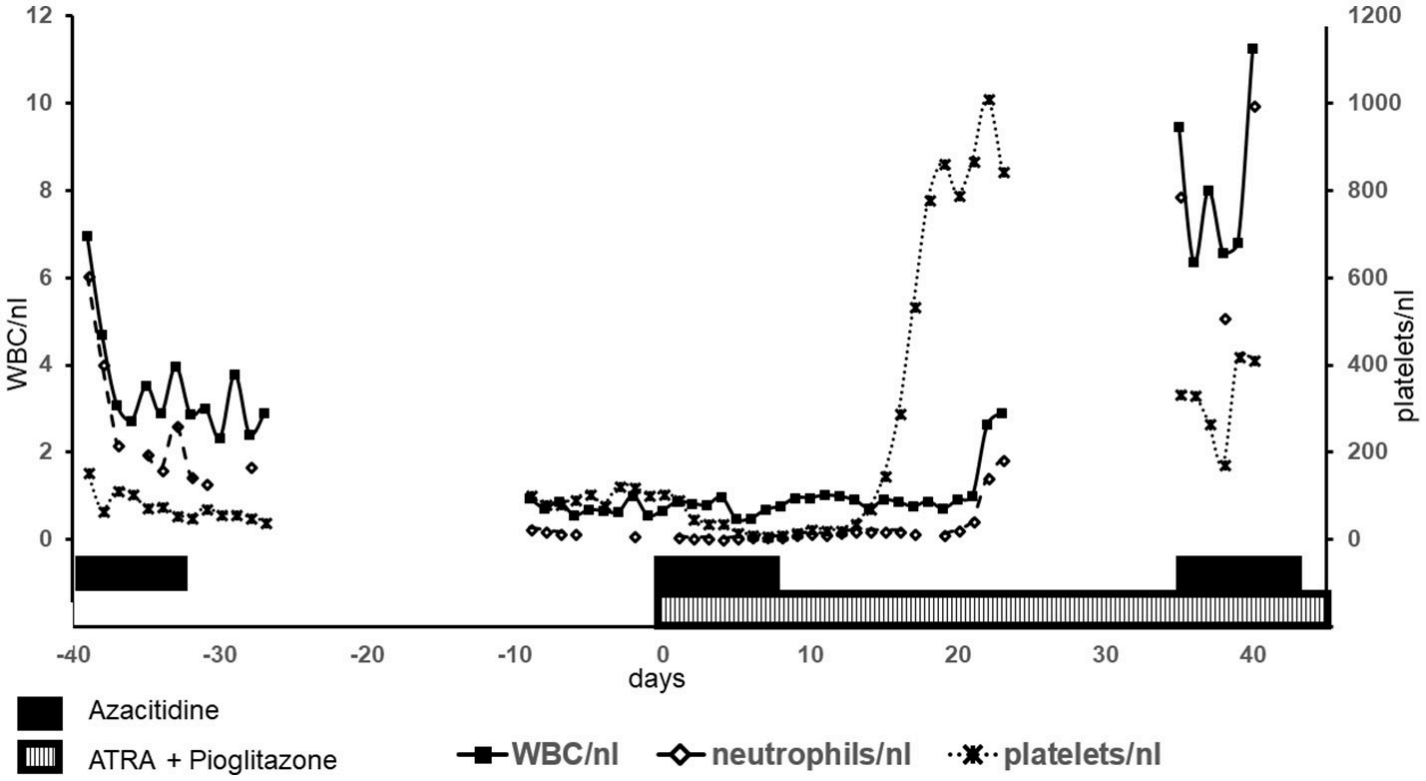
Blood counts: Blood counts of leukocytes (WBC), neutrophils and platelets are shown. Left y-axis shows values for WBC und neutrophils, right y-axis for platelets. The shown time period comprises the first cycle azacitidine as well as the first cycle of biomodulatory therapy (day 0 = begin of biomodulatory therapy).

After the successful diagnosis of MDS and LC a therapy with azacitidine (given sc 75 mg/m2 d 1–7 of 28) resembling the established first-line therapy in high risk MDS was started in September 2016. At the end of cycle 1 the patient developed severe cytopenia (leukocytes = WBC 0.94/nl, neutrophils 0.27/nl; Figure 2) with a percentage of 13% peripheral blasts (previously 0–2%) as well as fever without evidence for infection. While cytopenia alone could be just a common side effect of azacitidine, the combination of cytopenia, rising peripheral blasts and diminishing clinical condition of the patient (fever without evidence for infection) was interpreted as progressive disease refractory to hypomethylating agents (HMA). Due to rising peripheral blasts transformation to acute myeloid leukemia (AML) was suspected. Unfortunately, this was not confirmed by bone marrow biopsy. With very little therapeutic opportunities outside clinical trials HMA failure is associated with very poor prognosis (Montalban-Bravo et al., 2018). In this situation, the therapeutic regimen was discontinued. After initial cytoreduction with hydroxyurea (500 mg bid for 5 days) the therapeutic regimen was changed to a biomodulatory regimen, consisting of low-dose azacitidine 75 mg/day (given sc d1-7 of 28), pioglitazone 45 mg/day per os, and all-trans-retinoic acid (ATRA) 45 mg/m2/day per os, which already had shown impressive results in chemorefractory AML. Previously five elderly AML patients had been treated with this approach on a compassionate-use basis outside of a clinical trial in the absence of alternative therapeutic options leading to induction of myeloid differentiation in leukemia blasts and resulting in molecular remissions in 3 of 5 patients (Thomas et al., 2015).

After cycle 1 of this combined biomodulatory therapy, the presented patient showed hematologic recovery. Besides a mild anemia (hemoglobin 11.1 g/dl), she developed normal blood counts (Figure 2) with the absence of peripheral blasts. Moreover, the cutaneous leukemic infiltrates which had been unaffected by the azacitidine alone ameliorated tremendously after 2 cycles resulting in a complete remission of the skin lesions after cycle 6 (Figures 1C–F). Unfortunately, on day 1 of the planned 7th cycle of biomodulatory therapy the patient showed diminished peripheral blood counts (leukocytes 0.7/nl, hemoglobin 11.5 g/dl, thrombocytes 67/nl); therapy was stopped and bone marrow biopsy showed progressive AML (40% marrow blasts). Genetic work-up showed clonal evolution with complex aberrant karyotype and two independent mutations in *TP53*. The treatment was changed to decitabine 20 mg/m^2^ (given iv d1-5 of 28). After two cycles of decitabine the patient showed rising peripheral blasts up to 60%. Decitabine was discontinued (end of May 2017). A cytoreductive therapy with mitoxantrone iv 10 mg/m^2^ weekly was started. Despite these changes, the patient died of progressive disease in June 2017.

Nevertheless, the response to biomodulatory therapy in this case is very remarkable especially in the context of HMA failure. Concerning LC treatment strategies usually involve systemic as well as local therapy (i.e., radiotherapy, total skin electron beam therapy) (Bakst et al., 2011). Persistent complete remission of LC upon biomodulatory therapy alone is therefore very noteworthy.

Combined transcriptional targeting of peroxisome-proliferator-activated-receptor gamma (PPARγ) and the retinoic acid receptor (RAR) as well as treatment with azacitidine have been previously shown to induce myeloid differentiation and inhibit leukemic cell growth (Curik et al., 2012; Tabe et al., 2012; Faber et al., 2013). The combination of all three drugs probably leads to a synergistic effect. Since such approaches are also widely applicable in heterogenic (chemorefractory) metastatic diseases (Reichle and Vogt, 2008; Hart et al., 2016), the effect can be explained with the new therapeutic concept of “anakoinosis” which means reprogramming of communicative networks (Hart et al., 2015). Anakoinosis means re-establishing of tissue homeostasis by communicatively reprogramming transcriptional networks maintained by tumor and adjacent stroma cells. Therefore, the effects of “anakoinotic” treatments are dependent on the cellular context and the tumor-bearing organ. In the presented case the response to treatment was context-dependent as well (skin vs. bone marrow): it was possible to induce a persistent complete remission of leukemic skin infiltrates, while in the bone marrow (BM) “normal” hematopoiesis was transiently improved resulting in normal blood counts, but eradication of leukemic (stem) cells failed. In AML it is known that direct physical crowding of BM by accumulating leukemic cells does not fully account for hematopoietic failure (Boyd et al., 2017). In this context, the suppression of BM adipocytes plays an important role leading to imbalanced regulation of endogenous hematopoietic stem and progenitor cells, resulting in impaired myelo-erythroid maturation. PPARγ agonists have shown to induce BM adipogenesis, which rescues healthy hematopoietic maturation while repressing leukemic growth (Saiki et al., 2006; Boyd et al., 2017; Ryu et al., 2018). Targeting of the axis between adipogenesis und myelo-erythroid maturation might explain the hematologic improvement in our patient. Although an additional therapeutic agent had been needed to fully eradicate leukemic blasts (progressive disease is most likely due to clonal evolution in the BM), the transient improvement of hematopoiesis shows the effect of biomodulatory treatment on tissue homeostasis.

## Concluding remarks

In conclusion, we report a very unusual case with cutaneous infiltrates being the first clinical manifestation of hematologic disease, preceding the development of acute myeloid leukemia and therefore resembling a diagnostic challenge. While one cycle of azacitidine showed no effect on the skin lesions, a combined biomodulatory approach resulted in a complete remission of the cutaneous manifestation. Due to its efficacy in the shown cases the biomodulatory therapy is currently investigated within a prospective, randomized, clinical trial (AMLSG26-16/AML-ViVA EudraCT number 2016-000421-39).

## Methods

All genetics analyses (chromosome banding analysis, FISH und sequencing) have been performed within clinical routine diagnostics at MLL (Munich Leukemia Laboratory, Munich, Germany).

## Ethics statement

Written informed consent of the patient's husband (legal representative) for publication of the case was obtained.

## Author contributions

DH, JH, and AR treated the patient. DH and AR wrote the manuscript. All the authors revised the manuscript critically, approved the final manuscript, and agreed to be accountable for all aspects of the manuscript.

### Conflict of interest statement

The authors declare that the research was conducted in the absence of any commercial or financial relationships that could be construed as a potential conflict of interest.
